# Supplementary material for: Are carbon emissions trading and green financial instruments synergistic? -Comprehensive quantitative research based on content analysis
Source: PLoS One. 2024 Mar 7;19(3):e0298601. doi: 10.1371/journal.pone.0298601 (PMC10919601; doi:10.1371/journal.pone.0298601)
Supplement: S1 Data — (ZIP) [file pone.0298601.s001.zip › Data/地级市Z_F工作报告.zip!/║╙▒▒/╠╞╔╜╩╨/└┤╫╘╣1⁄2╓┌║┼í╛┬φ┐╦╩2╛▌═°í┐_2015.docx]

各位代表：

现在，我代表唐山市人民政府，向大会报告工作，请予审议，并请各位政协委员和列席会议的同志提出意见。

一、2014年政府工作回顾

过去的一年，是唐山发展历程中极不寻常、极不平凡的一年。我市经济遭遇了多年来少有的“冰冻期”，传统行业严重亏损，企业效益持续下降，部分经济指标增速创下近20年新低。全年完成地区生产总值6225.3亿元，增长5.1%；规模以上工业增加值3052亿元，增长4.6%；固定资产投资4146.2亿元，增长16%；一般公共预算收入323.7亿元，增长1.7%。全市经济正在经历一个历史罕见、异常严峻的艰难时期。一直以来，我市靠传统产业支撑发展，随着市场和形势的发展变化，“两高一低”问题越积越重。尤其是钢铁产能过剩导致市场低迷、效益下行等问题，从2012年下半年开始显现，2013年集中迸发，2014年全面扩散，迅速波及到其他行业，造成全市经济大幅下滑。钢坯价格由金融危机爆发前最高的每吨5600元，下跌到最近的2000元左右；铁精粉由2011年的每吨1510元跌至现在的640元；工业用电量增速骤然降至0.2%，为32年来的最低增幅。规模以上工业企业中，停产221家、亏损423家，分别占15.2%和29.2%。环保约束成为传统行业一条不可逾越的红线，两年来我市因环保问题关停取缔的企业达2382家。

冰冻三尺非一日之寒。我市经济之所以陷入如此被动境地，是多重因素叠加影响的结果。表象在于宏观经济形势和外部冲击所致。世界经济复苏一波三折、充满变数，我国经济发展进入中高速、多挑战的新常态，加之我们自身投资不旺、内需不振、消费不足，形成了经济持续下行的压力。根本在于长期积累的结构性矛盾集中凸显、相互交织。我市产业结构偏重趋同，产业链条短、产品档次低、竞争力不强，成为经济久提不振的根源所在。现在我们既面临全国“三期叠加”的普遍矛盾，又面临调结构、治污染、防风险的特殊压力，各种两难问题更加突出。关键在于产业青黄不接、转型阵痛加剧。一方面，压减过剩产能任务艰巨，对财税增收、民生就业以及相关行业的连带影响十分明显；另一方面，战略性新兴产业和现代服务业尚处在起步和成长阶段，还添不平秤、挑不起大梁，短期内难以对冲新旧转换造成的被动局面。核心在于发展方式尚未实现向创新驱动的根本转变。在资源环境约束加剧、传统比较优势弱化的情况下，我市的既有发展模式没有完全跟上新型工业化发展步伐，经济增长更多依靠传统生产要素的投入，内生动力明显不足，致使经济发展在困境中徘徊不前、难以打破僵局。

困难激发斗志，逆境催生力量。在极其严峻的特殊形势下，我们没有因为经济下行而失去振兴崛起的志气，没有因为遭受重创而减弱干事创业的劲头，没有因为爬坡过坎而放慢转型升级的步伐。全市上下在省委、省政府和市委的坚强领导下，坚定信心，迎难而上，统筹推进稳增长、调结构、促改革、治污染、惠民生，各项工作取得了新进展、新成效。一是结构调整稳中有进。全年实施亿元以上钢铁深加工项目107项，消耗本地钢材占粗钢产量的20.1%，提高5.6个百分点。工业技改投资1295.2亿元，增长14.2%。全面完成压减过剩产能年度任务。渤海钢铁联合重组进入实质性推进阶段。高新技术产业和装备制造业增加值增速分别达到20%和11.3%，高于工业增速15.4个和6.7个百分点。服务业完成投资2013.7亿元，占固定资产投资的48.6%。新增市级以上农业龙头企业90家，产业化经营率达到67%。二是沿海发展全面提速。沿海一线固定资产投资、一般公共预算收入分别增长19.2%和12.8%，高于全市3.2个和11.1个百分点。唐山港完成货物吞吐量5亿吨，跃升到全国第4位、全球第5位，集装箱首次突破100万标箱。曹妃甸协同发展示范区建设全面启动。临港产业聚集步伐加快，沿海基础设施和公共服务功能日趋完善，唐山工职院新校园正式开学，河北联合大学新校区建设主体基本完工。三是发展活力不断增强。国有企业、行政审批、公用事业、商事登记等重点领域改革取得积极进展。冀东发展等优势企业成功引进战略投资者，34家困难企业改制全面启动。新培育规模以上工业企业197家，新登记各类市场主体6.4万户，分别增长13.6%和18.7%。新增村镇银行5家、上市公司22家，燕赵财险正式开业。实际利用外资14亿美元，增长4.1%；进出口总额166亿美元，增长31%。四是城乡建设深入推进。世园会场馆、展馆、展园建设全面提速 完美世界小说。新建改造南新道等14条城市道路，城市二环路、唐古快速路开工建设。供热、供气、给排水等一批市政设施项目完工并投入使用。大剧院、图书馆、群艺馆等重点公共服务项目开工建设。农村面貌改造提升行动扎实推进，县城建设水平进一步提升。城镇化率达到56.2%，比上年提高1.2个百分点。五是环境质量总体好转。二氧化硫、二氧化氮、一氧化碳、臭氧、PM2.5和PM10分别下降36%、13%、11.1%、16.7%、12.2%和11.4%，空气质量达标天数增加29天，重度污染以上天数减少15天。六是人民生活持续改善。在经济形势严峻、财税增速下降的情况下，民生和社会事业支出保持了刚性增长。全年累计投入78.8亿元，占一般公共预算支出的74.1%。年初确定为群众办的十件实事，除京唐客运专线主体完工、待验收通车外，其余全部完成。城乡居民人均可支配收入分别达到28891元和12867元，增长8.2%和10.2%。信访、综治、维稳工作扎实有效，妥善处置化解了非法吸储和民间借贷等一批复杂矛盾和问题。安全生产和食品药品监管不断加强。教育、文化、卫生、体育等各项社会事业加快发展，民族宗教、外事侨务、广播电视、人口计生、妇女儿童、审计、统计、旅游、老龄、气象、档案、人防、编制、防震减灾等工作都取得了新的成绩。

在推进经济社会发展的同时，扎实开展党的群众路线教育实践活动，认真落实中央八项规定，坚决纠正“四风”方面的突出问题，干部作风发生明显变化。修订完善市政府工作规则，严格规范行政行为，市本级“三公”经费支出下降22.4%。自觉接受各方面监督，认真执行市人大及其常委会各项决议，办理人大代表建议207件、政协提案569件，按时办复率100%。

各位代表，通过一年来应对挑战、攻坚克难的实践，我们积累了经验，丰富了阅历，点燃了激情。这是当前最有价值的收获和最为宝贵的历练，为我们逆境图强、再创辉煌提供了坚实保障。这些成绩的取得，是省委、省政府和市委正确领导的结果，是全市人民团结拼搏、共同奋斗的结果。在此，我代表市政府，向全市人民，向人大代表、政协委员、各民主党派、工商联、无党派人士、人民团体和各界人士，向驻唐部队、武警官兵、公安政法干警，向关心支持唐山发展的老领导、老同志，向所有为唐山发展作出贡献的同志们、朋友们，表示崇高的敬意和衷心的感谢!

回顾过去的一年，我们也清醒地认识到，政府工作中还存在很多不足和薄弱环节，主要是：推动转型发展的思路、理念和方式方法还不能完全适应新形势、新任务的要求，攻坚克难、应对复杂局面的能力有待进一步提高；政府职能需要加快转变，社会管理和公共服务还存在薄弱环节；一些部门和工作人员缺乏担当精神，存在为官不为、懒政怠政现象，服务意识、办事效率与企业和群众的期待还有一定差距。对此，我们一定高度重视，采取有效措施，认真加以解决，决不辜负全市人民的厚望。二、奋力推动唐山转型升级、再创辉煌

当前，唐山发展已进入一个新的历史拐点，正在经历转型升级的剧烈阵痛。我们既面临困难，更有着千载难逢的发展机遇。省委、省政府对唐山的发展高度重视、寄予厚望。省委书记周本顺同志要求我们转观念、转作风、转方式，背水一战打好翻身仗。省长张庆伟同志要求我们探寻新常态下转型发展的新路径，努力为全省发展大局多做贡献。市委九届六次全会和全市经济工作会议明确提出，适应新常态，抢抓新机遇，推动唐山转型升级、再创辉煌，加快建设靓丽、繁华、宜居、和谐的综合性沿海强市。

历史经验表明，大到一个国家，小到一个城市，在其发展过程中都会经历若干兴衰成败的历史节点。在这样的重要关头，何去何从攸关前途命运。我们决不能让唐山这种被动局面再持续下去、蔓延下去了，必须坚定革新图变的决心，拿出扭转乾坤的新招，在推动发展的思维、理念、模式、路径、动力支撑上来一次大调整、大变革、大洗牌。唯有这样，才能从根本上摆脱困境，在沧海横流中抢抓新机遇、赢得新主动。今年和今后一个时期，政府工作的总体思路是：全面贯彻落实党的十八大、十八届三中、四中全会、中央和省经济工作会议精神，坚持稳中求进工作总基调，抢抓京津冀协同发展、环渤海地区崛起的新机遇，坚定不移地实施沿海开放带动战略，做大做强沿海增长极、中心城市和县域经济“三大经济板块”，抓实“九大增长点”，唱响“实干兴唐”主旋律，落实“四个干”机制，突出创新驱动，加速转型升级，保障改善民生，实现经济社会持续健康发展，如期全面建成小康社会，加快建设靓丽、繁华、宜居、和谐的综合性沿海强市。根据当前形势和我市发展现状，突出把握以下六大关键举措：

一是强力推动新兴产业和现代服务业加速崛起。这是转型升级的重要突破口，也是经济振兴的通天大道。要将新兴产业和现代服务业作为赢得未来竞争的“双引擎”，使其尽快成长为“参天大树”，早日挑起发展大梁。要大胆进军、深度开发新的领域。瞄准新能源、节能环保、生物医药、高端装备制造、电子信息等重点领域，以总投资850亿元的中核冀东(迁西)核电、331亿元的东华能源页岩气新材料、300亿元的协鑫集团LNG码头及综合利用、350亿元的汉能薄膜太阳能发电、500亿元的丰润动车城等重大支撑项目为龙头，填补空白，补齐短板，催生新的增长点。做大做强三产服务业。优先发展生产性服务业，提速壮大生活性服务业，依托总投资436亿元的中铁润驰北方物流等25个重点物流项目，总投资420亿元的华润万象城、勒泰城等15个重点商业综合体项目，总投资480亿元的唐山湾国际旅游岛等10大旅游项目，总投资45亿元的唐山金融中心和26亿元的路北医疗健康产业园区，加快构建起完备的现代服务业体系。通过实施大项目、好项目，开辟新领域、新产业，努力在区域竞争中转败为胜、换回主动。

二是力促传统产业脱胎换骨、既有企业东山再起。传统产业是我们现阶段的立身之本，既有企业是唐山发展的基石。在当前这个爬坡过坎的艰难时期，对传统产业要加大调整升级力度，做好“有中生新”的文章，不断催生出新产品、新技术、新产业。对既有企业要高看一眼、厚爱一层，决不能让他们在经济下行的“严冬期”坐冷板凳，感到没人管，被忽略。要从土地、信贷、市场、资金投向等方面制定具体的帮扶政策和措施，尤其是加大金融支持力度，引导既有企业积极开展二次创业，敢于打破旧的坛坛罐罐，赶赴新的战场。在全市推广渤海钢铁联合重组、减量搬迁的经验，推进天津物产对迁安鑫达、松汀、荣信3家钢铁企业的整合重组和股权改造，以此增强企业的生命力。坚持把钢结构产业化作为重大战略工程，大力引进发达国家的集成技术和设计、施工团队，推行工厂化生产、组装式施工，尽快把钢结构打造成唐山的领军产业，真正使我市的钢材有市场、有出路。加快实施投资68亿元的唐钢高强汽车板、投资50亿元的首钢迁钢特种钢基地等一批具有代表性的钢铁深加工项目，通过做实产业链、做强耗钢产业，使传统产业和既有企业焕发出新的生机和活力。

三是全力加快中心城市发展。城市建设的过程，就是培植税源、创造财富的过程 侯卫东官场笔记。2016年，我市既要承办世界园艺博览会，还将承办中国金鸡百花电影节、中国—中东欧国家地方领导人会议、中国—拉美企业家高峰会等。这对于加快城市现代化步伐，提升唐山在全国乃至世界的影响力、知名度和开放度具有重大意义。一方面，要通过世园会筹备工作，放大南湖生态城的生态、人文环境等优势，推进市中心区南拓，加快建设宜居、宜业、宜商、宜学的现代化城市，不断聚合各种生产要素，为财源建设提供支撑。另一方面，要发挥大型盛会的辐射带动作用，培育引进一批潜力大、前景好、创税多的项目，将城市打造成区域性商贸、金融、物流、消费、技术研发等财富聚集地。以路北“三城一中心”为龙头，谋划建设一批特色商业街区、大型商业综合体，以及总部经济、楼宇经济、金融服务、文化创意等方面的项目，推动城市经济扩规模、上水平。通过几年的努力，让唐山尽显现代都市的繁华景象，尽快打造成京津冀东北部的中心城市。

四是做大做强沿海经济增长极。目前，世界上90%的国际化大都市都是凭借沿海优势实现崛起的，当今世界十大经济中心几乎全部靠在沿海。唐山是海洋资源大市，兼具深水大港、燃气、电力、土地、综合保税区和国家级经济技术开发区等独特优势，经过多年的开发建设，沿海和港口已经到了发力阶段。必须打通并用好这个命脉和通道，围绕港产城一体发展，做活贸易，做大物流，做美港城，加强资源整合，加快构建“一港三区”的港口发展新格局，使沿海和港口成为名副其实的“聚宝盆”“摇钱树”。曹妃甸区、海港开发区主营业务收入、财政收入增速要保持在20%以上。尤其要按照“再造一个新唐山”的要求，推动曹妃甸开放开发实现大跨越、大提速，尽快成为全市乃至全省经济发展的领头羊、领跑者，不辜负省委、省政府和全市人民的厚望与重托。

五是依托京津实现借力发展。总书记亲自推动京津冀协同发展这一重大国家战略，目前已进入实质性运作阶段。对唐山而言，这是最大、最现实、最不可错失的机遇。尤其是北京与河北共同打造曹妃甸协同发展示范区，更加凸显了唐山在承接首都产业转移中的战略地位。全市上下、各行各业都要形成高度共识，积极行动、主动对接，着力抓好总投资2788亿元的中海油服新材料生产基地等141个重点项目，尽快让京津转移产业在唐山落地生根、开花结果。同时，放大京津冀协同发展的机遇，广泛借势造势，把世界的、全国的科技、人才、资金引到唐山，以此分享协同发展带来的巨大红利，提升我们的实力、活力和竞争力。

六是依靠创新创业打造新的增长点。出台支持创新创业的政策措施，集聚各种资源要素，激发全社会创造活力，催生新产业、新业态、新模式，让唐山的市场主体多起来、活起来。特别要下好科技创新的“先手棋”，制定实施科技型中小企业成长计划和鼓励扶持政策，力争通过3到5年的努力，培育打造以高新区为代表的10大科技园区，为科技型中小企业加快发展提供广阔空间；全市新增科技型中小企业5000家以上，使之成为新兴产业异军突起的开路先锋。只要我们在唐山这片沃土上掀起“大众创业”“草根创业”的新浪潮，形成“万众创新”“企业创新”的新局面，经济发展动力就能实现根本转换，可持续发展能力就会得到极大增强。

上述六个方面，既是发达国家和地区跨越发展的成功做法，也是资源型城市振兴崛起的必经之路。尽管新的征程充满挑战，但更面临着难得的“天时”、独特的“地利”，唐山发展的春天已经来临。我们坚信，通过全市人民的共同努力，唐山的各项事业一定会实现新跨越、再创新辉煌，一个靓丽、繁华、宜居、和谐的综合性沿海强市，一定会更加精彩地展现在世人面前!三、2015年主要工作任务

今年是全面完成“十二五”规划的收官之年，是全面深化改革的关键之年，也是我市转型升级、再创辉煌的重要一年。主要预期目标是：地区生产总值增长5%以上，一般公共预算收入增长5%以上，固定资产投资增长16%，社会消费品零售总额增长11%，进出口总额增长5%，实际利用外资增长5%。单位生产总值能耗降低率、主要污染物削减量和城市环境空气质量改善率完成省达目标要求。城乡居民人均可支配收入分别增长8%和8.5%，居民消费价格指数涨幅控制在3%左右，城镇登记失业率控制在4.5%以内，人口自然增长率控制在6.9‰以内。按照“抓实九大增长点”的要求，重点在以下十个方面实现突破：

(一)以招商为主抓手、开发区为主战场，着力在项目建设上实现突破。没有项目，就走不出低谷、扭转不了被动局面。今年，全市安排10亿元以上重点项目200个，年度完成投资1500亿元。一是坚持抓大不放小。集中优质资源要素，突出抓好一批引领转型升级、拉动税源增长、支撑一方发展的立市立县大项目。加快国泰纸业、正大通用焊管等140个续建项目建设进度，力促渤海钢铁、中石化千万吨级炼油、华润电厂二期、三友20万吨短纤维等30个投资20亿元以上项目的开工建设。同时，谋划实施一批周期短、见效快、效益好的短平快项目，新培育中小微企业1万家，新增市场主体3.5万户。二是全方位招商引资。打好“沿海”“京津”两张牌，面向长三角、珠三角、港台等重点区域，实施“大招商、招大商、招好商”，着力引进一批战略投资者。年内，突出抓好200个投资5亿元以上的重点招商项目。三是全面释放开发区的功能优势。进一步完善配套设施和服务功能，制定出台既合规又务实管用的扶持政策，全面强化考核，让开发区在全市发展中打头阵、当先锋、做表率，真正成为可依赖、能支撑的经济增长点。年内，各开发区固定资产投资要增长25%以上，主营业务收入和税收增速达到20%以上。每个开发区要新引进3至5个10亿元以上的产业项目。到年底，主营业务收入超100亿元的开发区达到19个以上，超500亿元的8个以上，超1000亿元的2个以上。

(二)坚持“有中生新、无中生有”两手抓、两手硬，着力在工业转型升级上实现突破。工业是唐山经济的脊梁和大厦，是税收、就业的根本依托。围绕推动传统产业脱胎换骨，科学搞好顶层设计，统筹做好加减乘除法，分门别类地研究新对策、制定新措施，着力在提质、增效、升级上下功夫，让“老树生新枝、结新果”。一是加快延伸产业链条。力促钢结构产业化迈出实质性步伐，抓好冀东超大型海上平台、金牛管材生产基地、滦州重工机械等100项投资亿元以上的钢铁深加工项目，加快打造10大耗钢产业基地，全市消耗本地钢材比重要达到25%以上。二是突出抓好技术改造。谋划实施工业技改项目500项，技改投资增长12%以上。重点引进开发无头轧制、智能机器人、物联网数控系统等40项新技术，抓好阻燃粘胶短纤维等100项新产品开发，推动产品由低端向中高端的跨越。三是有序化解过剩产能。通过严格的环保标准、实施差别化政策等措施，强力压减过剩产能和淘汰落后，全面完成省达我市的年度目标任务。四是全面加强企业管理。开展百家企业管理提升行动。学习借鉴国内外先进企业经验，从规模、效益、机制、市场等方面，广泛开展与同行业的对标，实施企业家素质提升工程，不断增强企业的内生动力。五是培育打造行业龙头。在全市筛选100家优势骨干企业予以重点扶持，实施动态管理，确保营业收入年均增长20%以上；新培育规模以上工业企业80家，支持冀东发展、唐钢集团、津西钢铁、轨道客车、三友集团等企业营业收入早日突破500亿大关、迈进“千亿俱乐部”。六是着力壮大战略性新兴产业。加大政策扶持力度，集中力量发展一批新产业，催生一批重点企业、优势产品和知名品牌，促其迈向高端化、集群化。抓好总投资1215亿元的轨道客车扩能、百川研发基地等132个重点项目，战略性新兴产业增加值增长10%以上。

(三)推动服务业大发展、快发展，着力在形成趋于合理的产业结构上实现突破。坚持多措并举、上下联动，力促服务业发展步入快车道、迈上新台阶。今年，服务业增加值确保增长8%以上。一方面，做大做强生产性服务业。加快发展现代物流、金融保险、信息服务等产业，促进工业与服务业高度融合发展。制定出台现代物流业发展规划，整合用好公路、铁路、港口等资源，大力发展第三方物流，抓好10大物流产业聚集区建设，新增入园企业300家，确保天物综合物流、恒业国际货运等15个重点项目完工投产。通过物流业的发展，降低企业经营成本，增强抗风险的能力。完善金融服务体系，力争村镇银行实现县域全覆盖，支持燕赵财险、唐山银行做强做大，全年新增上市公司10家以上。积极推进国家级信息消费试点城市建设，推动云计算、大数据、物联网在制造业领域的推广应用。发展壮大楼宇经济、总部经济，加快打造硅谷大厦、新华贸、高科总部大厦等一批纳税超千万、上亿元的商业写字楼。积极支持我市电子商务、服务外包等新兴业态发展，年内建成20个特色产业电子商务交易平台、2个省级以上电子商务产业园区。另一方面，全面提升生活性服务业。从规划、审批、用地、金融等方面制定扶持措施，改造提升商业购物、餐饮娱乐、社区服务等传统服务业。华润万象城、义乌小商品城、新华道地下商业街确保年内开工，勒泰城、香港新世界商贸中心年底前投入运营。引导支持社会资本进入教育、文化、体育、医疗卫生、健康养老等领域，大力发展检验检测中心等第三方医疗服务。年内，路北医疗健康产业园区建设取得实质性进展，市区启动建设2所示范性医养结合服务机构，谋划建设6个社会化养老基地，把服务老年人的“夕阳红”事业打造成蓬勃发展的朝阳产业。强化旅游资源整合，加快景区提档升级，推出一批精品旅游线路，旅游业总收入增长20%以上，早日把唐山建成独具特色、令人向往的旅游目的地。

(四)全面提速沿海经济发展，着力在打造国际物流大港上实现突破。继续举全市之力，聚焦沿海、会战沿海，使其尽快成为我市转型升级、跨越发展的强大引擎。一是加快完善港口功能。推动曹妃甸五、六号港池和丰南港区通用码头及早开工。年内唐山港货物吞吐量达到5.1亿吨以上，集装箱135万标箱。进一步做活做强做精六大宗商品交易，全面增强港口造血机能。充分释放综合保税区优势，着力引进一批高端管理人才和成长性好、竞争力强的外向型企业，谋划实施一批大宗过境商品就地深加工和物流项目，使其尽快发挥应有效应。二是加快临港产业聚集。今年，沿海地区实施亿元以上产业项目500项，完成投资1000亿元以上。力促华润电厂二期、协鑫集团LNG码头及综合利用等项目开工建设，确保汉能一期、中粮精炼糖、中硅联量子技术应用等项目完工投产，加快首钢二期、大型海水淡化等项目前期工作，推动沿海产业发展驶入快车道。抓紧谋划引进一批发挥临港优势、充满生机活力的中小项目，形成热火朝天、大干快上的生动局面。三是加快曹妃甸协同发展示范区建设。加强与北京合作，尽快完成试验区规划编制、开发主体组建、产业基金设立等工作，抓紧展开先行启动区基础设施建设。力促航天万源新能源产业园等12个已签约项目开工建设，推动福田10万辆重卡生产基地等重大项目尽快落地。四是加快港城建设步伐。围绕把唐山湾生态城、曹妃甸临港商务区做精做美，抓紧完善配套服务功能，放宽政策限制，引入民间资本，大力发展休闲娱乐、商务商贸、滨海旅游等服务业态，加快聚集人气、商气、财气，让曹妃甸真正热起来、火起来。全力提速河北联合大学新校区建设，确保明年寒假完成整体搬迁。统筹推进以唐曹、水曹、汉曹铁路为重点的集疏运体系建设，张唐铁路建成通车，力促迁曹高速、遵曹公路加快建设、早日投入使用，尽快形成内畅外联、四通八达的路网功能，为集聚产业和人流提供支撑。

(五)狠抓龙头带动和规模经营，着力在现代农业发展上实现突破。农业是万业之基，是永远的朝阳产业。坚持农业的基础地位不动摇，做大做强现代农业，培育新的经济增长点。一是发展壮大新型农业经营主体。围绕打造京津农副产品供应基地，新发展专业大户230个、家庭农场100个、农民专业合作社100家、达到市级以上标准的农业产业化龙头企业30家。培树一批新型主体典型，抓好蓝猫集团野生酸枣汁扩建等100个产业化重点项目。二是积极拓宽农业投融资渠道。落实各项惠农政策，创新涉农资金投入方式，鼓励工商资本投资适合企业化和工厂化发展的现代农业，抓好华农公司等31个超亿元的工商资本投资农业项目。试行农业“政银保”合作贷款，开展蔬菜温室大棚和生猪价格指数保险试点，鼓励农业企业上市融资和再融资。三是加快转变农业发展方式。制定标准化生产规程，启动市食品药品综合检验检测中心建设，积极创建农产品质量安全市。年内，新增市级以上农业标准园62个，标准化种植面积达到700万亩以上。抓好卧龙谷农科园等50个现代农业产业园区、燕东生态观光园等8个休闲农业示范园建设。加快冀东果菜批发市场、金古月国际绿色产品物流港等农产品产地市场和新型流通业态建设。完善农业基础设施，新发展节水灌溉面积20万亩，建设高标准农田10万亩。强化基层农技服务功能，年内培训新型职业农民5000人。四是着力提升改善农村人居环境。加强山水林田湖生态修复，实施矿山生态治理、绿化攻坚等重点工程，新增造林37万亩，耕地应用配方施肥520万亩。深入开展农村面貌改造提升行动，对350个省级重点村进行综合整治，让农村成为农民安居乐业的美丽家园。

(六)深入实施创新驱动战略，着力在增强转型发展内生动力上实现突破。坚持以科技创新为引领，扭住重点行业、骨干企业和关键技术，以创新推动转型升级迈出实质性步伐。一是培育激活企业创新主体。抓好企业技术中心、机器人产业技术研究院、高新区创业中心、中国农科院唐山粮油试验站等一批创新平台建设。年内，新建省级以上企业技术中心10家、市级以上重点实验室4个、工程技术研究中心20个以上，争创省级院士工作站2个，新增科技型中小企业450家、小巨人企业25家，高新技术产业增加值增长12%。二是加快推进科技研发和成果转化。深入实施钢铁、装备制造等七大科技创新专项，加强高品质特种钢等一批关键技术的研发推广，加快科技大市场和科技服务大厦建设。全年实施重点科技转化项目50项以上，发明专利申请量和授权量分别增长10%以上。三是深化与京津的协同创新。进一步强化与京津高校、科研院所的合作，抓好曹妃甸中关村高科技产业园区、北京硅谷国际科技产业园区建设，落实与中国农大的科技战略合作协议，推进芦台开发区、汉沽管理区与天津共建创新型产业示范园区，全面提升我市的创新能力。四是大力强化人才支撑。完善人才引进的政策措施和评价办法，设立曹妃甸沿海人才改革试验区，吸引更多的优秀人才来唐创新创业。大力发展现代职业教育，打造一支适应转型发展需要的复合型、应用型、技能型产业大军。年内，新增特邀院士10名，培养引进高技能人才2万名。

(七)深化改革开放，着力在释放发展活力上实现突破。改革开放是经济社会发展的活力之源。改革方面，重点是把国有企业、行政体制、财税金融等领域改革不断推向深入。国企改革：探索组建国有资本运营公司，由管资产向管资本转变。力促唐山港集团等企业引进战略投资者，发展混合所有制经济。加快推进34家困难企业盘活存量资产，实现有序退出。行政体制改革：启动实施新一轮市、县政府机构改革和简政放权。严格机构编制管理。推进开发区管理体制改革。抓好曹妃甸区行政审批“一章制”改革试点工作。6月底前在全市范围内实现网上审批。财税金融体制改革：实行全面规范、公开透明的预算管理制度，深化绩效预算管理改革。加快金融发展集团组建工作，设立产业投资引导基金。推行PPP模式，拓宽融资渠道。加强地方政府债务管控，依法规范民间融资管理。社会事业改革：推进市政公司等生产经营性事业单位改革，健全政府向社会力量购买服务制度，完成机关公车制度改革。扎实推进市级公立医院改革，确保上半年取得实质性进展。农村综合改革：规范农村墓地规划管理。加强农村宅基地管理。建立县级农村产权流转交易市场，推进土地承包经营权确权登记颁证，到年底，累计完成承包地总面积的60%。启动实施供销社综合改革。土地供应制度改革：探索试行租赁使用、短期出让、先租后让等土地供应政策措施，有效破解项目用地瓶颈制约。扩大开放方面，树立大开放思维，努力在提升经济外向度上实现新突破。加强出口基地建设，优化出口商品结构。以唐钢、冀东发展等骨干企业为龙头，加快传统优势行业“走出去”步伐，建立境外生产制造基地，支持有实力的企业到境外实施并购、上市、投资建厂、开展先进技术及劳务合作，拓展新的发展空间。(八)全面加快以世园会筹办为重点的城市建设，着力在提升城市功能、品位和形象上实现突破。以靓丽、繁华、宜居、和谐为目标，集中力量打一场城市建设总体战、攻坚战。对世园会场馆、展馆、展园建设，倒排工期，挂图作战，确保年内全部竣工。加快实施会址周边道路、市政配套和景观提升工程，力促早日完工。一是统筹推进城市基础设施建设。新建、翻修国防道等21条城区道路，老205国道改造、唐古快速路8月底前通车，城市二环路年底前完工，京沈二通道年内开工，火车站西广场全面竣工，构建内外贯通、高效便捷的城市交通体系。启动实施陡河电厂供热改造和大型调峰锅炉建设，推进南湖水厂、丰润污水处理厂扩建改造等市政设施项目，提升城市的承载能力。二是着力完善城市功能。加快教育、文化、卫生、商业等公共服务设施建设，抓好文化广场、奥体中心、科技馆、工人文化宫、新青少年宫等重点项目，高标准规划建设一批餐饮、娱乐、商务、休闲设施，打造一批独具魅力的“城市会客厅”。三是全面提升城市形象。大力实施“绿美亮净”综合整治，重点抓好8条城市主干道和5个出入口的景观提升，搞好临街建筑立面改造，建设一批具有唐山特色、体现工业文明、富有现代气息的标志性建筑和精品示范街，确保城市的每个节点都是靓点。推进“智慧唐山”建设，推行“数字化”城管新模式。开展严重超限超载车辆集中整治。搞好城市停车场的规划建设。深化全国文明城市创建工作。四是抓好县城和小城镇建设。按照一定的规模要求，每个县城和中心镇推进一批供热、给排水、垃圾处理等基础设施，谋划实施一批公共服务项目。每个县至少培育3个特色产业集群，重点把县城周边的产业园区做强做精，实现以产兴城、产城互动。筛选一批基础条件较好的建制镇予以重点支持，培育一批工业强镇、商贸重镇、旅游名镇和现代农业特色镇。五是加快推进农业转移人口市民化。科学设定进城落户办理流程，抓好社保、医疗、就学等政策衔接，实现“同城同权利、同城同待遇”。年内，城镇化率要达到57.4%。

(九)持续开展环保治理攻坚，着力在改善生态环境质量上实现突破。以新《环保法》为统领，以更大力度抓好各项治理和防范措施的落实，为全市人民创造一个良好的生态环境。一是加快重点行业污染治理。围绕全面实现企业达标排放，高标准实施钢铁、水泥等五个行业的脱硫、脱硝、除尘和堆场改造，对市区周边钢铁企业探索推行“球团直接入炉”生产工艺。加快市区及周边污染企业退城入园和搬迁改造，全面启动唐山电厂、新兴焦化、征楠焦化等7家企业的停产或搬迁工作。二是狠抓燃煤压减。深入推进燃煤锅炉整治，确保年底前原煤散烧锅炉全部退出主城区供热历史。大力推广洁净型煤，年内市区和县城全面禁止散烧原煤和高硫煤。加快推进“气化唐山”工程，实现管道天然气“县县通”。三是加强环境综合整治。大力推广餐厨垃圾无害化处理。加大各类干散物料堆场和道路扬尘治理力度，全部取缔城市环线内露天堆场和混凝土搅拌站。对环保整治不达标的矿山企业坚决关停取缔。四是加大水污染治理力度。制定出台水污染防治行动计划，开展焦化企业地下水污染专项整治。加强潘家口、大黑汀、邱庄、陡河水库等重点库区的环境治理，谋划启动管道引水项目，让市民喝上安全水、放心水。狠抓青龙河、还乡河等重点河流的综合整治，努力实现河畅其流、水复其清。五是强化环保监管。对7个区重点污染企业全部安装空气质量监测装置，实行全天候监测。对各类环境违法行为要依法严厉打击。

(十)加强民生保障和社会建设，着力在提升人民群众生活质量上实现突破。百姓利益无小事,民生问题大于天。要始终不渝地把改善民生作为一种责任和追求,既要普遍关心，又要高度关怀，使唐山成为最有人情味、最具幸福感的城市。今年，市财政安排民生和社会事业资金79亿元，占一般公共预算支出的76%。一是抓好就业这个民生之本。启动职业技能公共实训中心建设，妥善做好化解过剩产能过程中职工分流安置工作，统筹解决好高校毕业生、退役军人、城镇困难人员、农村转移劳动力等重点人群就业问题，确保城镇新增就业9万人以上。二是抓好社保这个民生之依。实施社会保障扩面提质工程，加大贫困家庭救助力度，提高残疾人帮扶救助水平，推动慈善事业健康发展。三是抓好教育这个民生之基。引进北京优质教育资源，加快改造义务教育薄弱学校，实施第二期学前教育三年行动计划，启动开滦一中搬迁建设，唐山幼专确保通过省验收。四是抓好就医这个民生之急。完善公共卫生服务体系，加强城乡基层医疗卫生服务能力建设，市中心医院、新妇幼医院年内投入使用。五是抓好文化这个民生之魂。进一步完善城乡公共文化服务体系，加快唐山陶瓷创意中心、金土地文化娱乐中心等20个文化产业项目建设。六是抓好安全稳定这个民生之根。深入推进“平安唐山”“法治唐山”建设，高度重视和加强信访稳定工作，探索推行以网格化管理为重点的社会治理新模式，完善立体化社会治安防控体系。加大食品药品安全监管力度，狠抓安全生产管理，确保人民群众饮食用药和生命财产安全。加强国防动员和民兵预备役建设，做好人口计生、民族宗教、外事侨务、广播电视、妇女儿童、老区建设、防震减灾、气象、档案等各项工作。

各位代表，改善民生，只有起点，没有终点。今年，我们将继续为群众办好十件实事。一是进一步提高城乡低保标准和医保最高支付限额，增加基础养老金待遇，降低“特检特治”个人自付比例。二是财政投入资金，为市中心区困难群体提供保障性住房1000套。三是市动物园和植物园建成向社会开放。四是持续改善环境质量，新建空气质量监测站27座，淘汰黄标车6.2万辆，购置环保公交车200部。五是全面启动饮水安全提升工程，加强水源地综合治理，谋划启动管道引水项目，让市民喝上安全水、放心水。六是改造建设标准化唐山殡仪馆。七是深入实施农村环境综合整治提升工程。八是启动建设西南交通大学唐山国际学院。九是唐曹铁路、水曹铁路全面开工建设，打通曹妃甸至北京的快速通道，为方便群众出行和降低企业运输成本提供保障。十是文化广场建成投入使用，为人民群众提供更加优质的公共文化服务。

四、凝聚起攻坚克难、实干兴唐的强大合力

做好今年工作，推动转型升级、再创辉煌，政府责任重大。我们要以改革创新的精神加强政府自身建设，提振精气神、汇聚正能量、力促新跨越。

要尽心尽责、勇于担当。越是困难越要担当。全体政府工作人员特别是各级领导干部要增强忧患意识，时刻牢记责任和使命，在其位、谋其事、负其责、尽其力，重任之下敢担当，困难面前不回避，坚决摒弃“为官不易、为官不为”和不求有功、但求无过等错误思想，把全部心思和精力投入到干事创业上来。深入开展不作为专项整治行动，集中根治懒政、庸政、怠政、误政。我们在工作上允许失误、宽容失败，但绝不容忍不干事、不作为、不担当。

要只争朝夕、真抓实干。坚持以实干论英雄，凡事都要立足早、着眼快、突出实、追求好，做到快谋划、快上手、快实施，以最短的时间获得最大的成效。尤其要按照“四个干”抓落实机制，实行政府工作目标管理责任制，做到工作有部署、有督导、有考核、有结果。全体政府工作人员都要经常扪心自问，这一天、这一月、这一年，自己为唐山发展干成了哪些事、增了什么光、添了多少彩，以实际行动让实干兴唐的主旋律响彻唐山大地。

要改进服务、优化环境。进一步解放思想、转变观念，唱响“投资到唐山、事事都好办”，以开明开放的气度和胸襟服务发展、促进发展，真正把投资者当亲人、当朋友，讲感情、讲诚信、讲法治，多设路标、少设路障，确保各类投资主体在唐山一路畅通、一路绿灯、和谐满意。从严惩处破坏发展环境的行为，彻底解决“门好进、脸好看，但事还是难办”的问题，遇事快办办好，一切从简去繁，使唐山真正成为机制活、体制顺、成本低、效率高的投资热土和服务高地。

要依法行政、从严治政。坚持法定职责必须为、法无授权不可为，依法履行好经济调节、市场监管、社会管理和公共服务职能。建立重大决策合法性审查机制，推行政府法律顾问制度，保证依法决策、民主决策、科学决策。全面加强政务公开工作。认真贯彻执行市人大及其常委会的决议决定，自觉接受人大、政协以及各界群众和新闻媒体的监督，保障和支持审计、监察等部门依法行使监督权。狠抓教育实践活动整改落实，以中央八项规定为准则，坚决反对和克服“四风”。厉行勤俭节约，反对铺张浪费，严控“三公”经费支出。扎实推进反腐倡廉建设，把从严管理干部作为一种新常态，加强行政权力电子监控体系建设，强化对土地出让、政府采购、国有资产产权交易、建设工程招投标等重点领域的监管，严厉查处各类违法违纪案件，坚决纠正损害群众切身利益的不正之风，打造为民、务实、清廉的政府形象。

各位代表，勤劳智慧的唐山人民在改革发展的大潮中，创造了一个又一个辉煌。当前，唐山正站在一个新的历史起点上，面临着前所未有的机遇和挑战。狭路相逢勇者胜。只要我们拿出逢山开路、遇水搭桥的智慧和勇气，咬定青山不放松，不达目的不罢休，就一定能够战胜各种艰难险阻，唐山的发展就会迎来一个崭新的时代。让我们紧密团结在以习近平同志为总书记的党中央周围，在省委、省政府和市委的坚强领导下，同心同德，拼搏奋进，为加快建设靓丽、繁华、宜居、和谐的沿海强市而努力奋斗!
